# Genome-Wide Identification, Evolution, and Female-Biased Expression Analysis of Odorant Receptors in *Tuta absoluta* (Lepidoptera: Gelechiidae)

**DOI:** 10.3390/life14070872

**Published:** 2024-07-12

**Authors:** Cong Huang, Xiaolan Ou, Yusheng Wang, Yanan Zhou, Guifen Zhang, Wanxue Liu, Fanghao Wan, Hongbo Jiang, Yibo Zhang

**Affiliations:** 1 Key Laboratory of Entomology and Pest Control Engineering, College of Plant Protection, Southwest University, Chongqing 400716, China; huangcong@caas.cn (C.H.); oxl123123@outlook.com (X.O.); 2 State Key Laboratory for Biology of Plant Diseases and Insect Pests, Institute of Plant Protection, Chinese Academy of Agricultural Sciences, Beijing 100193, China; yushengwang01@163.com (Y.W.); 15315469602@163.com (Y.Z.); zhangguifen@caas.cn (G.Z.); liuwanxue@caas.cn (W.L.); wanfanghao@caas.cn (F.W.)

**Keywords:** *Tuta absoluta*, odorant receptors, female-biased expression, phylogeny, tandem duplication

## Abstract

The tomato leafminer, *Tuta absoluta* (Lepidoptera: Gelechiidae), is a highly destructive invasive pest targeting Solanaceae crops. Its olfactory system plays a crucial role in host location, mate finding, and other behavioral activities. However, there is a notable gap in the literature regarding the characterization of its chemosensory genes. In this study, we conducted a genome-wide identification of 58 odorant receptors (ORs) of *T. absoluta.* The identified ORs exhibit coding sequence (CDS) lengths ranging from 1062 bp to 1419 bp, encoding proteins of 354 to 473 amino acids. Gene structure analysis showed that the majority of these ORs consist of five, seven, eight, or nine exons, collectively representing 67% of the total ORs identified. Through chromosomal mapping, we identified several tandemly duplicate genes, including *TabsOR12a*, *TabsOR12b*, *TabsOR12c*, *TabsOR21a*, *TabsOR21b*, *TabsOR34a*, *TabsOR34b*, *TabsOR34c*, *TabsOR62a*, and *TabsOR62b*. The phylogenetic analysis indicated that six *TabsORs* were clustered within the lepidopteran sex pheromone receptor clade, while an expansion clade containing ten *TabsORs* resulted from tandem duplication events. Additionally, five *TabsORs* were classified into a specific OR clade in *T. absoluta*. Furthermore, through RNA-Seq and RT-qPCR analyses, we identified five *TabsORs* (*TabsOR21a*, *TabsOR26a*, *TabsOR34a*, *TabsOR34c*, and *TabsOR36*) exhibiting female-antennae-biased expression. Our study provides a valuable foundation to further investigations into the molecular and ecological functions of *TabsORs*, particularly in relation to oviposition behavior. These findings provide foundational data for the future exploration of the functions of female-biased expression OR genes in *T. absoluta*, thereby facilitating the further development of eco-friendly attract-and-kill techniques for the prevention and control of *T. absoluta*.

## 1. Introduction

Insects rely heavily on their chemosensory system to locate mating partners, host plants, food, and oviposition sites. This system also aids in the identification of toxic compounds, evasion of natural enemies, and facilitation of communication [1,2]. Consequently, chemical communication plays a vital role in insect dispersal, reproductive success, and the extent of the damage they inflict. Central to this process are the odorant receptors (ORs), a crucial gene family within the insect chemosensory system. Insect ORs are composed of a conserved coreceptor (ORco) and a diverse array of odorant receptors (ORx). These receptors form the ORx–ORco complex, which acts as a ligand-gated cation channel in olfactory sensory neurons. This complex is responsible for transducing the chemical signals to electrical signals, ultimately eliciting corresponding behavioral responses [3].

The chemosensory mechanisms of female and male insects exhibit notable distinctions. In many insect species, males rely on olfaction to detect and respond to the sex pheromones released by females, facilitating mate finding. Conversely, females predominately locate appropriate oviposition locations by detecting host plant volatiles [4]. This sexual dimorphism extends to the differential expression profiles of OR genes between female and male insects [5]. Across various insect orders, a large number of male-biased PRs have been deorphanized and are primarily responsible for the perception of female sex pheromones. Examples include *AlucOR4* of *Apolygus lucorum* in the Hemiptera [6], *SfruOR13* and *SfruOR16* of *Spodoptera frugiperda* in the Lepidoptera [7], *CchlOR18* and *CchlOR47* of *Campoletis chlorideae* in the Hymenoptera [8], *HparOR14* of *Holotrichia parallela* in the Coleoptera [9], and *MdesOR115* of *Mayetiola destructor* in the Diptera [10].

Conversely, the expressions of female-biased ORs predominately respond to host plant volatiles and are often associated with egg-laying behaviors. Instances of such ORs have been documented in *Aedes aegypti* [11], *Aethina tumida* [12], *Bactrocera dorsalis* [13], *Campoletis chlorideae* [14], and *Anastatus japonicus* [15]. In moths, female-biased ORs also play a role in detecting oviposition signals. For instance, in *Helicoverpa assulta*, the oviposition behavior regulated by host plant volatiles is mediated by two female-biased odorant receptors, *HassOR67* and *HassOR31* [16,17]. Similarly, in *Helicoverpa armigera*, the female-specific odorant receptor *HarmOR56* mediates oviposition deterrence [18]. Comparable observations have been made in *Plutella xylostella* [19] and *Manduca sexta* [20].

The tomato leafminer *Tuta absoluta* (Meyrick, 1917) (Lepidoptera: Gelechiidae), native to Peru, South America, at present, has emerged and inflicted damage in more than 100 countries globally [21] and poses a significant threat to the sustainability and productivity of the tomato industry. When infestations occur, they can lead to significant losses in tomatoes, ranging from 80% to 100% of the yield [22]. Although insecticides have historically been the primary method of control, their heavy and frequent use has contributed to the development of resistance in *T. absoluta* populations [23,24]. As a more environmentally friendly alternative, attract-and-kill strategies have garnered attention. However, their efficacy is limited by factors such as the polyandrous mating behavior of males and the high fertility rates of females [25]. Consequently, there is an urgent need for the development of attractants targeting both sexes, with a particular emphasis on attracting females.

Previous studies have identified numerous volatile compounds from host plants that elicit the electrophysiological responses in the antennae of female *T. absoluta* [26,27,28]. However, the specific compounds capable of attracting females remain unclear. In recent years, a reverse chemical-ecology-based approach has been employed to discover attractants and repellents. This approach involves identifying and screening active compounds by elucidating the functions of odorant receptors [29,30,31,32,33]. However, the current understanding of the types, quantities, and expression patterns of the odorant receptors in *T. absoluta* is limited. This knowledge gap significantly impedes the development of female attractants.

In the present study, we conducted a comprehensive genome-wide analysis of OR genes, with a specific focus on identifying the female-biased expression ORs in *T. absoluta*. A total of 58 ORs were successfully identified, and their gene structures and chromosomal locations were systematically analyzed. Subsequently, we constructed a phylogenetic tree incorporating the OR genes from four lepidopteran insects (*Bombyx mori*, *Helicoverpa armigera*, and *Spodoptera littoralis*) to explore their evolutionary relationships. Additionally, we utilized transcriptome data to calculate the expression profiles of ORs across different tissues in female and male adults. Furthermore, the high expression levels of the identified ORs in the antennae of female adults were validated by a real-time reverse transcription-polymerase chain reaction (RT-qPCR) analysis. Our findings provide valuable insights into the evolutionary dynamics of OR genes in *T*. *absoluta* and provide essential data support for future endeavors aimed at developing female attractants.

## 2. Materials and Methods

### 2.1. Identification of OR Genes in T. absoluta

The protein sequences of ORs from three lepidopteran insects (*B. mori*, *S. littoralis*, and *H. armigera)* were collected from the published articles in which these ORs had been previously identified from their respective genomes [34,35,36]. These sequences were utilized as queries in iterative BLASTP searches against *T. absoluta* genomes [37], using an e-value threshold of 1 × 10^−5^. Subsequently, candidate OR genes were subjected to a local command line HMMER (version 3.1b2) search against the Pfam-A database, specifically targeting the 7tm_6 (PF02949) or 7tm_4 (PF13853) hidden Markov model (HMM) profiles for further validation. 

### 2.2. Gene Structure Analysis and Chromosomal Location of OR Genes

The Generic Feature Format Version 3 (GFF3) format file containing all OR genes was initially extracted and subsequently submitted to the online web servers GSDS 2.0 for visualization and drawing of gene structures. Additionally, an in-house Python script (https://github.com/jackiexls/ChrLocPlotter, accessed on 10 June 2022) was used to generate distribution maps depicting the chromosomal locations of OR genes.

### 2.3. Phylogenetic Analysis

To investigate the evolutionary relationship of OR genes between *T. absoluta* and other lepidopteran moths, a total of 217 OR genes from four species (58 from *T. absoluta*, 54 from *B. mori*, 45 from *H. armigera*, and 60 from *S. littoralis*) were included in the phylogenetic analysis. Multiple sequence alignment of the protein sequences was performed using MAFFT v7 [38] with default parameters, followed by trimming of the alignment sequences using trimAl v1.2 [39] with the parameter “-automated1”. A maximum likelihood evolutionary tree was constructed using RAxML (version 8.2.12) [40], with the best-fit model (JTT + G + F) estimated with ProtTest3 software v3.4.2 [41]. Visualization and labeling of the phylogenetic tree were conducted using FigTree software v1.4.3 (http://tree.bio.ed.ac.uk/software/figtree/, accessed on 10 June 2022) and Adobe Illustrator CC 2017.

### 2.4. Expression Profiles of 58 TabsORs in Female and Male Antennae

The expression levels of 58 *TabsORs* in the antennae of female and male adults were determined using transcriptome data (unpublished). Paired-end clean reads were mapped to the *T. absoluta* genome using HISAT2 version 2.2.1 [42]. Fragments per kilobase of transcript per million mapped reads (FPKMs) were calculated using StringTie version 2.1.7 software [43]. Subsequently, the FPKM values were transformed to log2(FPKM + 1) and utilized as inputs for generating a histogram in Excel 2021.

### 2.5. RT-qPCR Verification of the Candidate Female-Antennae-Biased Expression ORs

Antennae samples from female and male adults (1 to 2 days old) were collected and immediately snap-frozen in liquid nitrogen before storage at −80 °C. Total RNA was extracted using a Total RNA extraction Micro kit (Genstone Biotech, Beijing, China). The reverse-transcription reaction was performed using a Hifair^®^ III 1st Strand cDNA Synthesis SuperMix for qPCR (gDNA digester plus) (Yeasen Biotechnology, Shanghai, China). In the first step, 15 μL of reaction mixture including 1 μg of total RNA, 3 μL 5×gDNA digester Mix, and RNase-free H_2_O was used to remove residual genomic DNA contamination in RNA templates. In the second step, a total of 20 μL of reaction mixture including 15 μL of reaction mixture from the first step and 5 μL 4×Hifair^®^ III SuperMix Plus was used to perform the reverse transcription and generate the cDNA.

The resulting cDNA templates were diluted 10-fold with ddH_2_O, and 0.5 μL of the diluted cDNA was used as template for subsequent RT-qPCR analysis. RT-qPCR was conducted on an ABI 7500 Real-Time PCR System (Applied Biosystems, Foster City, CA, USA) with Hieff™ qPCR SYBR^®^ Green Master Mix (Low Rox Plus) (Yeasen, Shanghai, China) to quantify the relative expression of *TabsOR* genes. RT-qPCR reactions were performed in a total volume of 10 µL, comprising an initial denaturation step at 95 °C for 5 min, followed by 40 cycles of denaturation at 95 °C for 10 s, and extension at 60 °C for 34 s, with a dissociation curve analysis. The process for each sample was performed in three duplicates. The relative expression levels of *TabsOR* genes were determined using the 2^−∆∆CT^ method and normalized by ribosomal protein L5 (RPL5) expression. All primers used are listed in Supplementary Table S1.

### 2.6. Statistical Analysis

The expression levels of both the FPKM values and RT-qPCR results were analyzed by Student’s *t*-test. Data are presented as the mean ± standard error (mean ± SEM). Differences were considered statistically significant at *p* < 0.05 and highly significant at *p* < 0.01.

## 3. Results

### 3.1. Identification and Gene Structure Analysis of OR Genes in T. absoluta

A comprehensive search for the OR genes in *T. absoluta* was conducted using 159 OR genes from *B. mori*, *H. armigera*, and *S. littoralis* as the queries in a BLASTP search (e-value < 1 × 10^−5^) against the genome of *T. absoluta*. Subsequently, the best BLAST hits were used to identify the conserved domains using hidden Markov models (HMMs) through a hmmscan search against the Pfam database. A total of 58 *TabsOR* genes harboring the 7tm_6 (PF02949) or 7tm_4 (PF13853) HMM profile were successfully identified. The coding sequence (CDS) lengths of the 58 *TabsOR* genes ranged from 1062 bp to 1419 bp, encoding proteins consisting of 354 to 473 amino acids (Supplementary Table S2).

**Figure 1:**
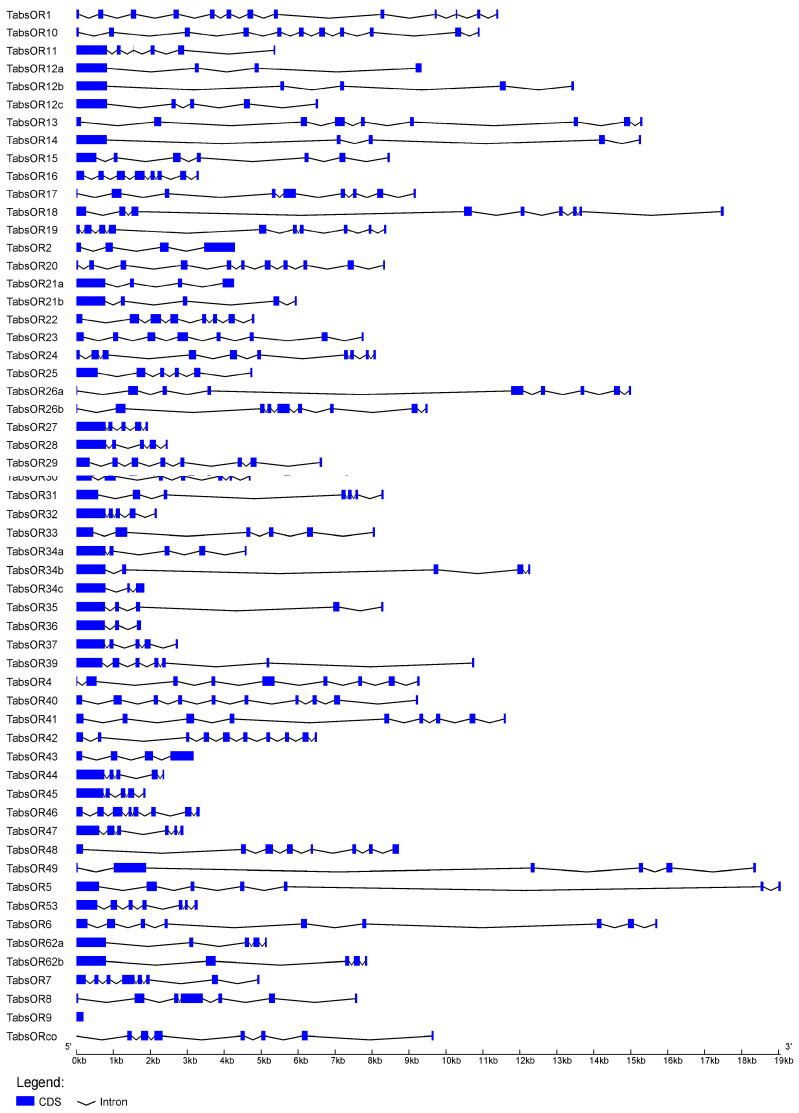
Gene structure of *TabsOR* genes. Blue boxes represent exons, while black lines represent introns.

The analysis of the gene structures of the *TabsOR* genes revealed variation in the number of exons, ranging from 1 to 13. Notably, *TabsOR9* has only one exon structure, while *TabsOR34c* and *TabsOR36* possess three exons each. Furthermore, four genes (*TabsOR12a*, *TabsOR2*, *TabsOR21a*, and *TabsOR3*) have four exons, fifteen genes (*TabsOR12b*, *TabsOR12c*, *TabsOR14*, *TabsOR21b*, *TabsOR27*, *TabsOR28*, *TabsOR32*, *TabsOR34a*, *TabsOR34b*, *TabsOR35*, *TabsOR37*, *TabsOR44*, *TabsOR45*, *TabsOR62a*, and *TabsOR62b*) have five exons, five genes (*TabsOR11*, *TabsOR25*, *TabsOR33*, *TabsOR47*, and *TabsOR49*) have six exons, eight genes (*TabsOR15*, *TabsOR30*, *TabsOR31*, *TabsOR39*, *TabsOR5*, *TabsOR53*, *TabsOR8*, and *TabsORco*) have seven exons, eight genes (*TabsOR16*, *TabsOR22*, *TabsOR23*, *TabsOR29*, *TabsOR3*, *TabsOR46*, *TabsOR48*, and *TabsOR7*) have eight exons, eight genes (*TabsOR13*, *TabsOR17*, *TabsOR18*, *TabsOR26a*, *TabsOR26b*, *TabsOR4*, *TabsOR41*, and *TabsOR6*) have nine exons, four genes (*TabsOR19*, *TabsOR24*, *TabsOR40*, and *TabsOR42*) have ten exons, and two genes (*TabsOR10* and *TabsOR20*) have eleven exons. *TabsOR1* displayed the most complex structure, with 13 exons (Figure 1).

### 3.2. Chromosome Location of TabsOR Genes

**Figure 2:**
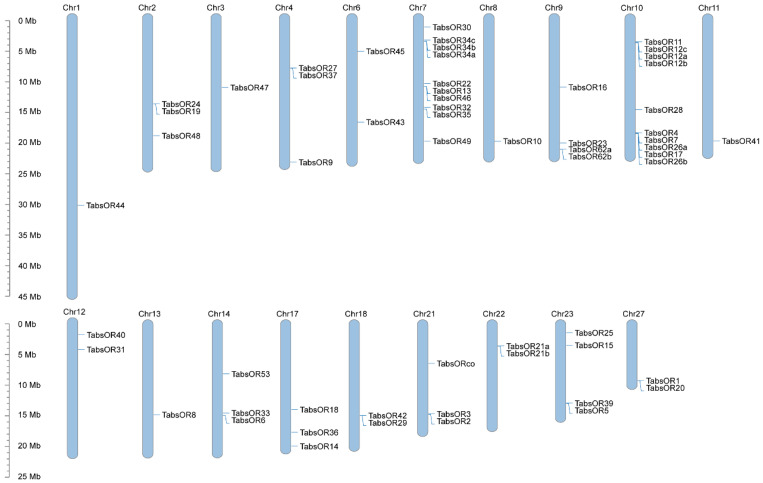
Chromosomal mapping of *OR* genes in *T. absoluta*.

All 58 *TabsOR* genes were mapped to the chromosomes of *T. absoluta*. The distribution of the *TabsOR* genes across the genome revealed that they are located on 19 different chromosomes (Figure 2). Notably, individual chromosomes exhibit varying numbers of *TabsOR* genes. Chromosomes 1, 3, 8, 11, and 13 each harbor a single *TabsOR* gene, while chromosomes 6, 12, 18, 22, and 27 contain two *TabsOR* genes each (Figure 2). Additionally, three *TabsOR* genes are located on chromosomes 2, 4, 14, 17, and 21, and four *TabsOR* genes are located on chromosome 9 and 23, respectively (Figure 2). Chromosomes 7 and 10 exhibit the highest number of *TabsOR* genes, with ten *TabsOR* genes located on each chromosome (Figure 2).

### 3.3. Phylogenetic Analysis of ORs

**Figure 3:**
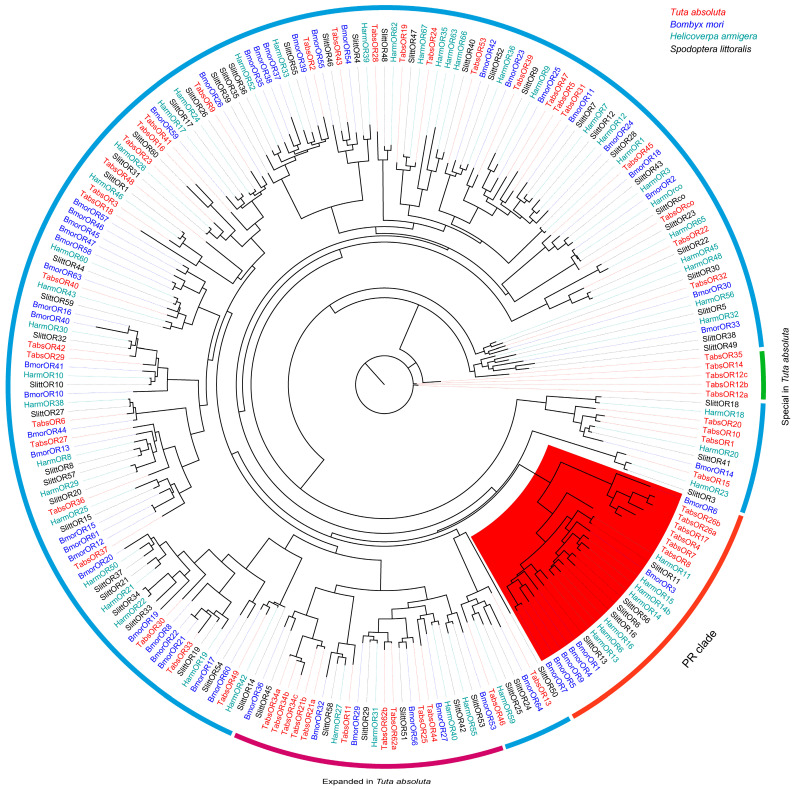
Phylogenetic tree of ORs in four lepidopteran species. For the tip labels: ORs in *T. absoluta* are labeled in red, those in *B. mori* are labeled in blue, those in *H. armigera* are labeled in green, and those in *S. littoralis* are labeled in black. For the branch clades: the red-shaded clade is a sex pheromone clade.

To explore the evolutionary relationship of the OR genes in *T. absoluta* with those of other lepidopteran species, a phylogenetic analysis was conducted using a dataset comprising 217 OR genes from *T. absoluta*, *B. mori*, *H. armigera*, and *S. littoralis*. The resulting phylogenetic tree revealed distinct clades representing different evolutionary lineages of OR genes. A subset of OR genes from *T. absoluta*, including *TabsOR4*, *TabsOR7*, *TabsOR8*, *TabsOR17*, *TabsOR26a*, and *TabsOR26b*, clustered together in a well-defined clade corresponding to the lepidopteran sex pheromone receptor clade (Figure 3). This clustering pattern suggests a shared evolutionary history and functional specialization related to sex pheromone perception in these genes.

Another notable observation was the presence of expanded OR gene families specific to *T. absoluta*, as evidenced by the clustering of genes such as *TabsOR34a*, *TabsOR34b*, *TabsOR34c*, *TabsOR21a*, *TabsOR21b*, *TabsOR11*, *TabsOR62a*, *TabsOR62b*, *TabsOR25*, and *TabsOR44* (Figure 3). This expansion of the OR gene families may reflect adaptations to specific ecological niches or evolutionary pressures unique to *T. absoluta*. Furthermore, a distinct clade composed of *TabsOR35*, *TabsOR14*, *TabsOR12a*, *TabsOR12b*, and *TabsOR12c* genes was identified, suggesting the formation of a specific OR clade within *T. absoluta* (see Figure 3). The presence of this clade may indicate the functional divergence or specialization of these OR genes in *T. absoluta* compared to other lepidopteran species.

### 3.4. Expression of TabsOR Genes in Antennae of Female and Male Adults

**Figure 4:**
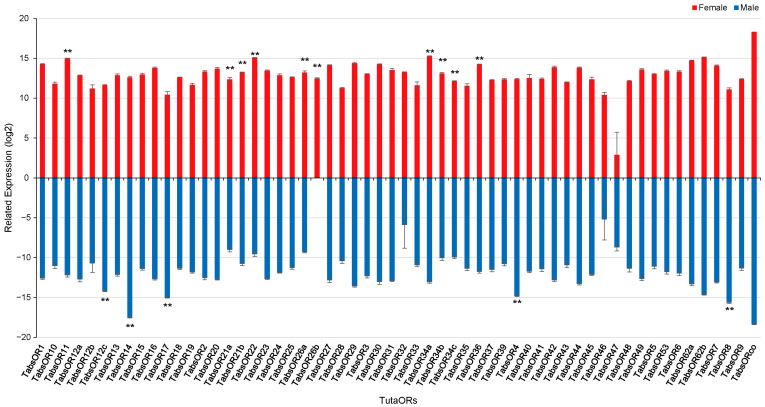
Expression levels in FPKM for *TabsOR* genes in the antennae of female and male adults. Asterisks indicate highly significant differences between mated females and males (** *p* < 0.01) (Student’s *t*-test).

The expression levels of the 58 *TabsOR* genes were assessed using the RNA-Seq data, allowing for a comparative analysis of the gene expression between female and male antennae tissues. The log-transformed fragments per kilobase of transcript per million mapped reads (log2(FPKM + 1)) values were utilized to facilitate the comparison of *TabsOR* gene expression levels. The analysis revealed significant differences in *TabsOR* gene expression between female and male antennae. Specifically, the genes exhibited significantly higher expression levels in female antennae than in male antennae. These genes include *TabsOR11*, *TabsOR21a*, *TabsOR21b*, *TabsOR22*, *TabsOR26a*, *TabsOR26b*, *TabsOR34a*, *TabsOR34b*, *TabsOR34c*, and *TabsOR36* (Figure 4). Conversely, five *TabsOR* genes had higher expression levels in male antennae, including *TabsOR12c*, *TabsOR14*, *TabsOR17*, *TabsOR4*, and *TabsOR8* (Figure 4). 

### 3.5. RT-qPCR Analysis of Candidate Female-Antennae-Biased Expression ORs

**Figure 5:**
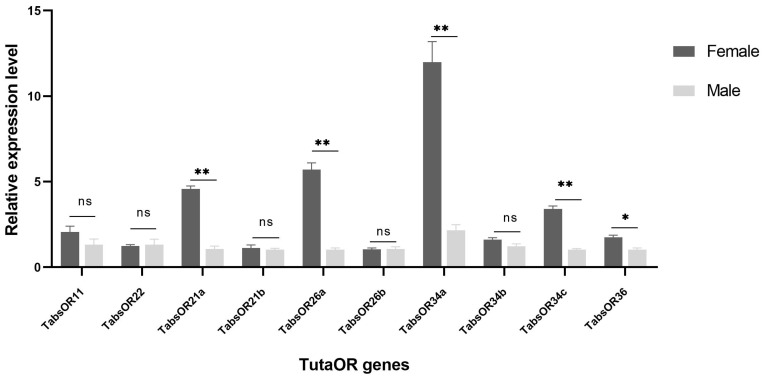
Expression profile of *TabsOR* genes in the antennae of female and male adults of *T. absoluta*. Asterisks indicate significant differences between mated females and males (* *p* < 0.05, ** *p* < 0.01, ns: not significant) (Student’s *t*-test).

To validate the differential expression of the *TabsOR* genes observed between female and male antennae and to corroborate the findings from the RNA-Seq analysis, we selected the ten OR genes exhibiting high expression levels in female antennae for an RT-qPCR analysis. The results showed that the expression levels of *TabsOR21a*, *TabsOR26a*, *TabsOR34a*, *TabsOR34c*, and *TabsOR36* were consistent with the RNA-Seq data, exhibiting significantly higher expression in female antennae compared to male antennae (Figure 5). Conversely, no significant differences in the expression levels were observed for *TabsOR11*, *TabsOR22*, *TabsOR21b*, *TabsOR26b*, or *TabsOR34b* between the female and male antennae (Figure 5).

## 4. Discussion

Insects rely heavily on their acute olfactory system to locate food sources, potential mates, and suitable oviposition sites, with OR genes playing a crucial role in these olfactory processes. Therefore, a better understanding of the olfactory system as well as the female-biased ORs in *T. absoluta*, a highly invasive and destructive pest, could contribute to the development of more effective and sustainable pest control strategies, such as attract-and-kill techniques or other environmentally friendly control methods. Despite the crucial role of OR genes, the understanding of this gene family remains limited in *T. absolute*. In this study, we conducted a comprehensive analysis of the OR gene repertoire in *T. absoluta* using genomic and transcriptomic data. Previous research has highlighted substantial variations in the numbers and sequences of insect OR genes, attributed to their ability to recognize the diverse odor signals present in different ecological habitats. The identification of 58 OR genes in *T. absoluta* represents a significant step toward elucidating the molecular basis of olfaction in this pest species. The observed number of OR genes in *T. absoluta* is similar to the number observed in other insect species; for instance, it is comparable to the OR gene count in *B. mori* (54 ORs), *Spodoptera exigua* (53 ORs), and *S. littoralis* (60 ORs). However, it deviates from the counts observed in certain insect species, such as *Cydia pomonella* (85 ORs) and *Spodoptera frugiperda* (69 ORs), while being greater than the count in *H. armigera* (45 ORs).

Understanding the gene structure is crucial for exploring the evolutionary relationships and functional diversity of OR genes. In the current study, we analyzed the intron-exon organization of *TabsOR* genes, revealing a range of one to thirteen exons. Notably, genes with five coding exons were the most abundant, comprising a total of 15 OR genes, followed by genes with seven, eight, or nine coding exons. Previous research has demonstrated that the gene structure can have a significant effect on gene function [44,45], suggesting the potential for both the functional conservation and diversity of the *TabsOR* genes.

The chromosomal distribution of genes can provide valuable insights into their evolutionary relationships and functional characteristics. Previous studies have demonstrated that the OR genes in insects are often arranged in tightly clustered formations on chromosomes, predominantly as a result of tandem duplication events. This clustering suggests potential functional similarities among the duplicated genes [45,46,47,48,49]. For example, in *C. pomonella*, the tandemly duplicated OR genes *CpomOR3a* and *CpomOR3b* on chromosome 17 exhibit synergistic and complementary functions in the recognition of host plants and mates [49]. 

In our study, we identified five tandemly duplicated OR gene clusters in *T. absoluta.* Specifically, *TabsOR34a*, *TabsOR34b*, and *TabsOR34c* are tandemly arranged on chromosome 7, while *TabsOR62a* and *TabsOR62b* are in tandem on chromosome 9. Additionally, *TabsOR12a*, *TabsOR12b*, and *TabsOR12c* form a tandem cluster on chromosome 10, along with *TabsOR26a* and *TabsOR26b*. Moreover, *TabsOR21a* and *TabsOR21b* are tandemly duplicated on chromosome 22. The similar research results in other insects indicate that tandemly duplicated OR genes play an important role in the adaptation to their specific environmental conditions [49]. Therefore, our results suggest that these tandemly duplicated TabsOR genes may play crucial roles in odor recognition, potentially contributing to the species’ capacity to adapt to its environment.

Previous studies have indicated that the orthologous genes clustered within the same clade often exhibit similar biological functions. To explore the evolutionary relationships and functions of *TabsOR* genes, we selected OR genes from the well-characterized species *B. mori*, *S. littoralis*, and *H. armigera* to reconstruct the phylogenetic tree [34,35,36]. Our analysis revealed that six *TabsOR* genes—*TabsOR4*, *TabsOR7*, *TabsOR8*, *TabsOR17*, *TabsOR26a*, and *TabsOR26b*—were cluster in the sex pheromone receptor clade, suggesting their potential involvement in sex pheromone recognition. In another clade, the *TabsOR* genes have exhibit expansion due to the tandem duplication of several genes, including *TabsOR34a*, *TabsOR34b*, *TabsOR34c*, *TabsOR21a*, *TabsOR21b*, *TabsOR11*, *TabsOR62a*, *TabsOR62b*, *TabsOR25*, and *TabsOR44*. Previous studies have suggested that the ORs within this clade (Clade K) are rapidly evolving OR genes with well-characterized functions. For instance, in *B. mori*, *BmorOR29* serves as a receptor for (±)-linalool, while *BmorOR56* detects (*Z*)-jasmone [34]. In *H. armigera*, *HarmOR27* is the receptor of butyl salicylate, *HarmOR31* is the receptor of (*Z*)-3-hexenyl acetate, *HarmOR40* is the receptor of geranyl acetate, and *HarmOR55* is the receptor of (*E*)-nerolidol [36]; in *S. littoralis*, *SlitOR29* is the receptor of ocimene [35]. The results suggest that the *TabsOR* genes within this clade may have a broad binding spectrum, indicating their potential roles in detecting diverse odorants. Furthermore, we identified a specific cluster of *TabsOR* genes comprising *TabsOR35*, *TabsOR14*, *TabsOR12a*, *TabsOR12b*, and *TabsOR12c*, suggesting their association with the unique biological characteristics of *T. absoluta*.

The expression patterns of genes are intricately linked to their functions [50]. In the case of insect ORs, the sex-biased expressions of ORs in antennae are very common. Typically, male-biased ORs are involved in detecting the sex pheromones released by females [8], whereas the female-biased ORs are associated with recognizing oviposition-related odors. Through the analysis of antenna transcriptome data and RT-qPCR detection, we identified *TabsOR21a*, *TabsOR26a*, *TabsOR34a*, *TabsOR34c*, and *TabsOR36* in the female-biased expression of ORs. It is worth noting that there is a large deviation in the expression trend of the *TabsOR26b* gene between the RNA-Seq and RT-qPCR results; we inferred that the reason may be that the sequences of the *TabsOR26a* and *TabsOR26b* genes are too similar. Because RNA-Seq relies on the ability to uniquely map reads to the genome or transcriptome, when genes are highly similar, reads can be incorrectly assigned to one gene or the other, leading to biased expression estimates. Finally, we speculated that the female-biased expression of OR genes may be involved in the detection of oviposition-related odor. However, further studies should be performed to determine the detailed functions of the identified OR genes in mediating specific behaviors such as mating and oviposition.

## Data Availability

All data described in the current work are available through access to the text or Supplementary Materials.

## Supplementary Materials

The following supporting information can be downloaded at: https://www.mdpi.com/article/10.3390/life14070872/s1, Table S1: Sequences of primers used for RT-qPCR studies; Table S2: Sequences of TabsOR genes.
